# Vldlr overexpression causes hyperactivity in rats

**DOI:** 10.1186/2040-2392-3-11

**Published:** 2012-10-30

**Authors:** Keiko Iwata, Nobuo Izumo, Hideo Matsuzaki, Takayuki Manabe, Yukiko Ishibashi, Yukio Ichitani, Kazuo Yamada, Ismail Thanseem, Ayyappan Anitha, Mahesh Mundalil Vasu, Chie Shimmura, Tomoyasu Wakuda, Yosuke Kameno, Taro Takahashi, Yasuhide Iwata, Katsuaki Suzuki, Kazuhiko Nakamura, Norio Mori

**Affiliations:** 1 Research Center for Child Mental Development, Hamamatsu University School of Medicine, Hamamatsu, Japan; 2 Department of Clinical Pharmacology, Yokohama College of Pharmacy, Yokohama, Japan; 3 Division of Gene Expression Mechanism, Institute for Comprehensive Medical Science, Fujita Health University, Toyoake, Japan; 4 Department of Synthetic Organic Chemistry, Yokohama College of Pharmacy, Yokohama, Japan; 5 Institute of Psychology and Behavioral Neuroscience, University of Tsukuba, Tsukuba, Japan; 6 Department of Psychiatry, Hamamatsu University School of Medicine, Hamamatsu, Japan

**Keywords:** Hyperactivity, Neurodevelopmental disorder, Psychiatric disorder, Reelin, Transgenic rat, Vldlr

## Abstract

**Background:**

Reelin regulates neuronal positioning in cortical brain structures and neuronal migration via binding to the lipoprotein receptors Vldlr and Lrp8. Reeler mutant mice display severe brain morphological defects and behavioral abnormalities. Several reports have implicated reelin signaling in the etiology of neurodevelopmental and psychiatric disorders, including autism, schizophrenia, bipolar disorder, and depression. Moreover, it has been reported that *VLDLR* mRNA levels are increased in the post-mortem brain of autistic patients.

**Methods:**

We generated transgenic (Tg) rats overexpressing Vldlr, and examined their histological and behavioral features.

**Results:**

Spontaneous locomotor activity was significantly increased in Tg rats, without detectable changes in brain histology. Additionally, Tg rats tended to show performance deficits in the radial maze task, suggesting that their spatial working memory was slightly impaired. Thus, Vldlr levels may be involved in determining locomotor activity and memory function.

**Conclusions:**

Unlike reeler mice, patients with neurodevelopmental or psychiatric disorders do not show striking neuroanatomical aberrations. Therefore, it is notable, from a clinical point of view, that we observed behavioral phenotypes in Vldlr-Tg rats in the absence of neuroanatomical abnormalities.

## Background

Reelin, a large secreted glycoprotein, is critical for normal brain development [1-3]. During embryonic development, reelin is secreted by specialized Cajal-Retzius cells to form a highly laminated structure in the neocortex, hippocampus, and cerebellum [2,4-6]. The reelin signaling pathway involves two reelin receptors, very-low-density lipoprotein receptor (Vldlr) and low-density lipoprotein receptor-related protein 8 (Lrp8), and the intracellular adaptor protein, disabled homolog 1 (Dab1) [7-9].

A number of studies have reported that reelin signaling is involved in the dopaminergic system, especially the expression of dopamine receptors in the nucleus accumbens [10,11]. Moreover, considerable evidence has implicated nucleus accumbens dopamine in the regulation of locomotor activity [12-17]. Additionally, recent studies have demonstrated the necessity for reelin signaling in certain aspects of hippocampal synaptic function, the formation of some forms of mammalian memory, and cognitive function [18-26].

Several reports implicate reelin signaling in the etiology of neurodevelopmental and psychiatric disorders, including autism, schizophrenia, bipolar disorder, and depression [27-35]. Post-mortem studies have reported decreased levels of reelin and its message in patients with autism, schizophrenia, and bipolar disorder, with less consistent findings for depression [27,29,30,32,33,36]. The finding of reduced reelin levels in these disorders has prompted interest in the reeler mutant mouse, which has a spontaneous mutation in the reelin gene, as an animal model of neurodevelopmental and psychiatric disorders. Homozygous reeler mutant mice are characterized by ataxia, tremors, imbalance, and a reeling gait, associated with severe hypoplasia of the cerebellum and neuronal ectopia in laminated brain regions [2]. Owing to the severe gait defects, it is difficult to evaluate other behavioral phenotypes. Heterozygous mice do not show obvious defects: two detailed studies have reported no defects in the behavioral phenotype of heterozygous mice [37,38], although a subsequent study reported deficits in contextual fear conditioning [39].

Fatemi *et al*. reported that post-mortem *VLDLR* mRNA levels are increased in the brain of autistic patients [29]. Furthermore, it has been reported that psychotropic drug treatment changes Vldlr expression in the rat brain [40]. However, little attention has been given to increased Vldlr expression. In this study, we generated transgenic (Tg) rats overexpressing Vldlr, and examined their histological and behavioral features.

## Methods

All experiments were approved by the Committee on Animal Research of Hamamatsu University School of Medicine, Yokohama College of Pharmacy and University of Tsukuba. All experiments were performed in accordance with the Guide for Animal Experimentation at the Hamamatsu University School of Medicine, Yokohama College of Pharmacy and University of Tsukuba.

### Generation of Vldlr transgenic rats

**Figure 1:**
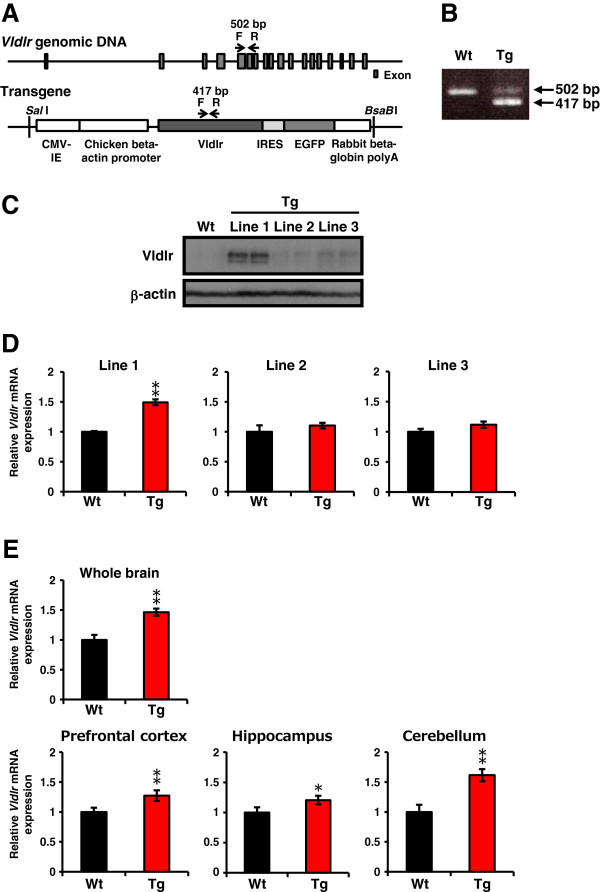
**Generation of Vldlr transgenic (Tg) rats.** (**A**) Map of *Vldlr* genomic DNA and the pCX-Vldlr-IRES-EGFP transgene. Full-length rat *Vldlr* cDNA was subcloned into the pIRES vector. The Vldlr internal ribosome entry site (IRES) was then subcloned into the pCX-EGFP vector containing the CMV immediate-early enhancer (CMV-IE)/chicken β-actin promoter and enhanced green fluorescent protein (EGFP). Arrows show positions of the PCR primers used to distinguish genomic DNA from the transgene. (**B**) PCR-based genotyping of transgenic rats. The primers identified a 502-bp wild-type (Wt) genomic DNA fragment and a 417-bp transgene (Tg) fragment. (**C**) Expression of endogenous (Wt) and transgenic (Tg) Vldlr protein in the liver at P0. Two pups from each line (1 to 3) were used. Vldlr from membrane fractions was detected by Western blotting using an anti-Vldlr antibody (upper panel). The blots were subsequently probed with an anti-β-actin antibody as a protein loading control (lower panel). The results are representative of three independent experiments. (**D**) qRT-PCR quantification of *Vldlr* mRNA expression in Wt and Tg whole brain at P0 (lines 1–3). (**E**) qRT-PCR quantification of *Vldlr* mRNA expression in Wt and Tg whole brain (upper panel), and prefrontal cortex, hippocampus and cerebellum (lower panels) in adult (2 to 3 months) rats from transgenic line 1. Relative *Vldlr* expression was obtained by normalizing to *Actb* from the same cDNA. Results are expressed as a ratio of Wt expression, resulting in a Wt ratio of 1. Error bars represent mean ± SEM (*n* = 3 to 6 per genotype). * *P* < 0.05, ** *P* < 0.01 between Wt and Tg rats.

**Figure 2:**
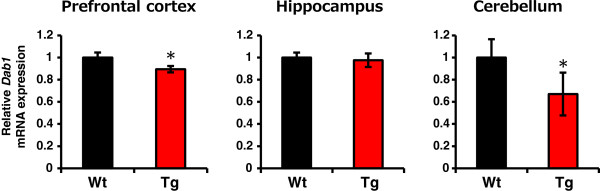
**Effect of Vldlr overexpression on *Dab1 *mRNA expression.** qRT-PCR quantification of *Dab1* mRNA expression in prefrontal cortex, hippocampus and cerebellum of wild-type (Wt) and transgenic (Tg) adult (2 to 3 months) rats from transgenic line 1. Relative *Dab1* expression was obtained by normalizing to *Actb* from the same cDNA. The results are expressed as a ratio of Wt expression, resulting in a Wt ratio of 1. Bars represent mean ± SEM (*n* = 6 per genotype). * *P* < 0.05 between Wt and Tg rats.

The full-length rat *Vldlr* complementary DNA (cDNA) was obtained from Dr. Masuzaki (University of the Ryukyus) [41]. The *Vldlr* cDNA was subcloned into the pIRES vector (Takara Bio, Inc., Otsu, Japan). The *Vldlr* internal ribosome entry site (IRES) was subsequently subcloned into the pCX-EGFP vector containing the CMV immediate-early enhancer (CMV-IE)/chicken β-actin promoter and enhanced green fluorescent protein (*EGFP*). The identity of the cloned gene was verified by DNA sequence analysis. Transgenic rats were generated by microinjection of a 6.3-kb Sal I/Bsa BI fragment (Figure 1A), from the expression vector pCX-Vldlr-IRES-EGFP, into the pronuclei of fertilized eggs. The injected embryos were transferred to pseudopregnant female Sprague–Dawley rats. Transgenic founders were identified by whole-body EGFP fluorescence and PCR using primers for *Vldlr* exon 5 (F: 5^′^-TTGTGTGCAATGGACAGGAT-3^′^) and exon 6 (R: 5^′^-CTTCATCAGAGCCGTCAACA-3^′^). This primer pair can distinguish the *Vldlr* transgene (a 417-bp fragment) from endogenous *Vldlr* (a 502-bp fragment) (Figure 1B). Three EGFP- and transgene-positive founders were obtained, but overexpression was confirmed in only one founder (Figures 2C,D). Thus, all studies were performed in a line derived from this founder. Unless otherwise stated, adult (2 to 3 months) rats were used for experiments.

### RNA isolation and quantitative real-time reverse-transcription-polymerase chain reaction

Total RNA was isolated from whole brain or specific brain regions, using TRIZOL Reagent (Invitrogen, Carlsbad, CA), and reverse transcribed using the SuperScript III First-Strand Synthesis System (Invitrogen). cDNA was used for qRT-PCR, which was performed using the SYBR GREEN PCR Master Mix (Qiagen, Hilden, Germany). *Actb* expression was used as a control for mRNA expression. Gene expression changes were quantified using the delta-delta C_T_ method. The primer sequences were as follows: *Vldlr* sense, 5^′^-TTCACATCCTCCATTCTCCA-3^′^; antisense, 5^′^-CAATCTCAATGATGCCCAAG-3^′^; *Dab1* sense, 5^′^-GCTTTGAAAGTCCCAGCAAG-3^′^; 5^′^-antisense, ATGGATCACTGGTGGAGGAG-3^′^; *Actb* sense, 5^′^-CGTGAAAAGATGACCCAGATCA-3^′^; antisense, 5^′^-AGAGGCATACAGGGACAACACA-3^′^.

### Membrane preparation

Rat livers were dissected in 0.9% NaCl at postnatal day 0 (P0). Tissues were placed in ice-cold homogenization buffer containing 0.32 M sucrose, 3 mM HEPES (pH 7.3) and a protease inhibitor Complete Mini tablet (Roche Diagnostics, Mannheim, Germany), for homogenization. Total lysates were centrifuged at 1,000*g* for 10 min at 4°C. The supernatants were collected and centrifuged at 20,000*g* for 30 min at 4°C. The pellets, containing the membrane fractions, were resuspended in 10% sodium dodecyl sulfate (SDS). The protein concentration in the membrane fractions was measured and normalized to 10% SDS.

### Western blot analysis

Membrane fractions (70 μg) were subjected to 7.5% SDS-polyacrylamide gel electrophoresis, followed by blotting to a polyvinylidene fluoride membrane (GE Healthcare, Little Chalfont, UK). The transferred membranes were incubated with a primary antibody against Vldlr (1:200; 6A6, Santa Cruz Biotechnology, Inc., Santa Cruz, CA) or β-actin (1:4,000; Abcam, Inc., Cambridge, MA) at 4°C overnight. After incubation, the membranes were washed three times with phosphate-buffered saline without Mg^2+^ or Ca^2+^ (PBS(−)), containing 0.1% Tween-20, and were then incubated with the appropriate secondary antibody (1:20,000) at room temperature for 1 h. The membranes were washed with PBS(−) containing 0.1% Tween-20, followed by visualization using an enhanced chemiluminescence system (ECL plus; GE Healthcare).

### Histology

Rats were anesthetized and perfused with 4% paraformaldehyde in PBS(−). The brains were removed and postfixed in the same fixative at 4°C, then transferred to 30% sucrose in PBS(−). When they sank, the brains were embedded in OCT compound (Tissue Tek, Sakura Finetek USA Inc., Torrance, CA) and frozen on powdered dry ice. Serial sections (30 μm) were cut and floated in PBS(−). The first series sections were stained with cresyl violet for Nissl staining. The second series sections were incubated overnight at 4°C with anti-neuronal nuclei (NeuN) antibody (mouse monoclonal IgG, 1:500; Millipore, Ltd., Watford, UK). The sections were then washed and incubated with biotin-conjugated anti-mouse IgG (1:200, Vector Laboratories, Burlingame, CA) for 1 h at room temperature. After washing, the sections were processed for 1 h using a Vectastain ABC kit (Vector Laboratories). Staining was visualized with diaminobenzidine (Vector Laboratories) as the chromatic agent. Control slices were incubated as described, but without primary antibody. No immunoreactivity was seen in the controls (data not shown).

### Behavioral tests

#### Open-field test

The open-field test is one of the best established ways to test exploratory locomotor activity in a novel environment. A square open field (90 cm × 90 cm) was constructed from gray polyamide enclosed by a 40 cm high surrounding wall. Each open field was divided into central (30 cm × 30 cm) and outer areas. The 15-min test was started after placing each rat individually in the same corner facing the wall. Behavior was recorded, and distance traveled (cm) and time spent in the central area (s) were analyzed, using the video-tracking system Viewer (version 2.2; Neuroscience, Inc., Tokyo, Japan).

#### Spontaneous locomotor activity test

To measure spontaneous locomotor activity, a rat was placed in a plastic cage (24.5 cm × 40 cm × 20 cm) with clean paper, food, and water. To ensure that the novelty of the cage did not confound the activity measurements, the first 17 hours of activity were not analyzed. Locomotion was measured per hour throughout the whole day using a digital counter with an infrared sensor (NS-AS01; Neuroscience, Inc.). The room light was on from 7:00 a.m. to 7:00 p.m.

#### Radial maze test

This test used an elevated eight-arm radial maze made of black polyvinyl chloride. The maze consisted of an octagonal central platform (32 cm in diameter) and eight arms (60 cm × 12 cm) radiating from the platform. A food well (1 cm in diameter, 0.5 cm deep) was carved out at the end of each arm. Plexiglas guillotine doors (15 cm high) divided the arms from the central platform, and each was operated automatically. The sidewalls of the arms were 4 cm high, except 12 cm from guillotine doors, where they were 12 cm high. The maze was elevated 70 cm above the floor. There were extra-maze visual cues (for example, a curtain, a desk, colored drawing paper, and a door) around the maze. Control and analysis of the behavioral experiment was carried out using Image RM (O’Hara Co., Ltd, Tokyo, Japan), a modified version of the free software NIH Image (National Institutes of Health, Bethesda, MD).

Rats were given 5 min handling for three days and then three daily sessions of habituation to the apparatus. In the habituation session, all the guillotine doors were opened and 20 mg food pellets (Research Diets, Inc., New Brunswick NJ) were placed on the platform and arms. In the first two sessions, five rats were placed in the maze together for 30 min, and in the last session, each rat was placed in the maze individually for 15 min. Rats were trained on the radial maze task for one trial a day. At the beginning of each trial, a 20 mg food pellet was placed in each food well. The rat was placed on the central platform and all the doors were opened. A choice was counted when the rat completely entered an arm; then all the doors except that of the chosen arm were closed. When the rat returned to the central platform, the door was closed and the rat confined there for 5 s. After that, all the doors were reopened and the rat was allowed the next choice. This procedure was repeated until the animal had consumed all the pellets, it had made 16 choices, or 10 min had elapsed since the start of the trial. A correct choice was defined when the rat entered an arm that had not previously been entered during the trial or in which the pellet had not been consumed; the other choices were counted as errors. The learning criterion was defined as five consecutive trials in which seven or more correct choices, in the first eight choices, were attained. The rats’ choice responses were recorded.

#### Social interaction test

Social interaction was tested in the same apparatus used for the open-field test. Each rat was tested for 15 min with a weight-matched partner from a different home cage. Social interaction was assessed by the time spent interacting, including sniffing, following, crawling over or under, grooming, and aggressive behaviors.

#### Elevated plus maze test

The elevated plus maze is a well-established model to assess the level of anxiety in rodents. The apparatus was made of gray polyamide and comprised two open arms (50 cm × 9 cm), two enclosed arms (50 cm × 9 cm × 50 cm), and a central platform (8.5 cm × 8.5 cm). The apparatus was elevated 50 cm above the floor. At the beginning of the test, the rats were placed on the central platform facing the same open arm. The test lasted 10 min and the behavior of the rat was recorded; the time spent in the closed and open arms was analyzed by a video-tracking system, as described.

#### Statistical analyses

Statistical significance of *Vldlr* and *Dab1* mRNA levels were analyzed using unpaired Student’s *t* tests, after it had been confirmed that there were no statistically significant differences in variance, as assessed by the *F* test. Repeated measures analyses of variance (ANOVA) and unpaired Student’s *t* tests were used for analysis of the behavioral tests.

## Results

### Generation of Vldlr transgenic rats

To evaluate the effects of increasing Vldlr expression in rats, we generated Tg rats harboring a Sal I/Bsa BI fragment from the expression vector pCX-Vldlr-IRES-EGFP (Figure 1A). Three transgenic founders were identified by PCR and whole-body EGFP fluorescence. The growth patterns of all founders and non-transgenic (wild-type, Wt) littermates were similar, indicating no severe effect of the Vldlr transgene in terms of growth of the Tg rats. All founders gave rise to true-breeding lines (lines 1, 2, and 3), and transmitted the transgene and EGFP fluorescence to their offspring. Both males and females reproduced at a rate similar to that of their Wt littermates, and their offspring grew normally.

First, we examined whether exogenous Vldlr was expressed at the plasma membrane in Tg rats. Vldlr is not expressed endogenously in rat liver [42]. Therefore, we collected membrane fractions from the livers of two pups, from each line, and examined Vldlr expression by Western blotting. Vldlr protein was strongly detected in line 1 pups, whereas the expression was very weak in line 2 and 3 pups (Figure 1C). Second, we confirmed overexpression of *Vldlr* mRNA in the whole brain by qRT-PCR. An approximately 1.5-fold increase in *Vldlr* mRNA was detected (Figure 1D) in line 1 Tg rats, compared with Wt rats, at P0 (*P* < 0.01). However, there was no significant increase in mRNA levels in lines 2 or 3. In line 1, *Vldlr* overexpression was detected in the adult (2 to 3 months) brain, at the same level as at P0 (*P* < 0.01; Figure 1E, upper graph). In addition, we measured *Vldlr* mRNA in multiple brain regions of relevance to autism, including the prefrontal cortex, hippocampus, and cerebellum, using adult rats. *Vldlr* expression levels were increased in these regions 1.3-, 1.2- and 1.6-fold, respectively, in Tg rats compared with Wt rats (*P* < 0.01, *P* < 0.05 and *P* < 0.01, respectively; Figure 1E, lower graphs). Thus, in transgenic line 1, Vldlr is stably overexpressed, including in brain regions relevant to autism. Finally, we investigated whether downstream signaling pathways are altered by Vldlr overexpression. The tyrosine residue of Dab1 is phosphorylated, following reelin binding to Vldlr [8]. It has been suggested that Dab1 phosphorylation causes a reduction in Dab1 protein levels via a negative feedback circuit [43]. Moreover, Fatemi *et al*. have reported increased *VLDLR* and reduced *DAB1* mRNA levels in post-mortem brain tissue (prefrontal cortex area 9 and cerebellum) from patients with autism [29]. Therefore, we measured *Dab1* mRNA expression in the prefrontal cortex, hippocampus, and cerebellum of adult rats. In Tg rats, *Dab1* expression levels were decreased in prefrontal cortex and cerebellum by 0.89- and 0.67-fold, respectively, compared with Wt rats (*P* < 0.05; Figure 2). However, there was no significant decrease in mRNA levels in the hippocampus, potentially driven by a reduced rate of Vldlr accumulation in hippocampus compared to prefrontal cortex or cerebellum. Overall, our results show that Vldlr overexpression changes downstream reelin signaling in Tg rats, at least in the prefrontal cortex and cerebellum. Moreover, Tg rats show abnormal expression of *Vldlr* and *Dab1* mRNA, as detected in post-mortem brain from patients with autism. Based on our expression results, we chose transgenic line 1 for further analysis.

### No abnormal brain morphology in Vldlr-Tg rats

**Figure 3:**
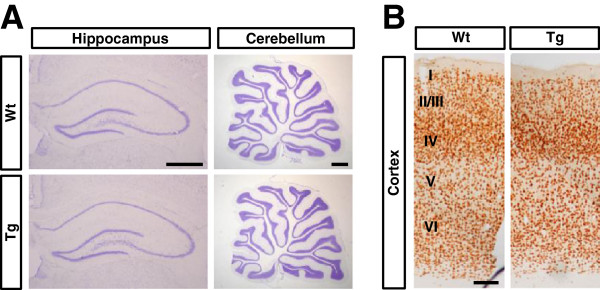
**Neuroanatomical characterization of Vldlr transgenic (Tg) rats.** (**A**) Histology of the hippocampus and cerebellum in adult (2 to 3 months) wild-type (Wt) and transgenic (Tg) rats. Panels show Nissl-stained coronal sections of the hippocampus or sagittal sections of the cerebellum. The results are representative of three independent experiments. Scale bars, 1 mm. (**B**) Cortical layering in adult (2 to 3 months) Wt and Tg rats. Panels show immunohistochemical NeuN staining. Cortical layers are numbered I to VI. The results are representative of three independent experiments (Additional file 3: Supplementary method). Scale bar, 100 μm.

Reelin regulates neuronal positioning in brain structures, including the neocortex, hippocampus and cerebellum. The effects of increased Vldlr on brain morphology have not previously been studied. We examined these brain structures in adult Tg rats, using Nissl staining (Figure 3A) and immunostaining with the neuronal marker, NeuN (Figure 3B). The neurons were layered normally in the neocortex and hippocampus, and the cerebellum was formed normally in Tg rats. Additionally, the number of NeuN-positive neurons and thickness of each cortical layer did not change significantly in Tg rats compared with Wt rats (Additional file 1: Figure S1). There were no abnormalities in other brain areas in which reelin signaling is not involved (data not shown). Moreover, there were no histological abnormalities in the neocortex, hippocampus, cerebellum (Additional file 2: Figure S2), or other brain areas examined (data not shown) at P0.

### Vldlr-Tg rats show hyperactivity and memory impairment

**Figure 4:**
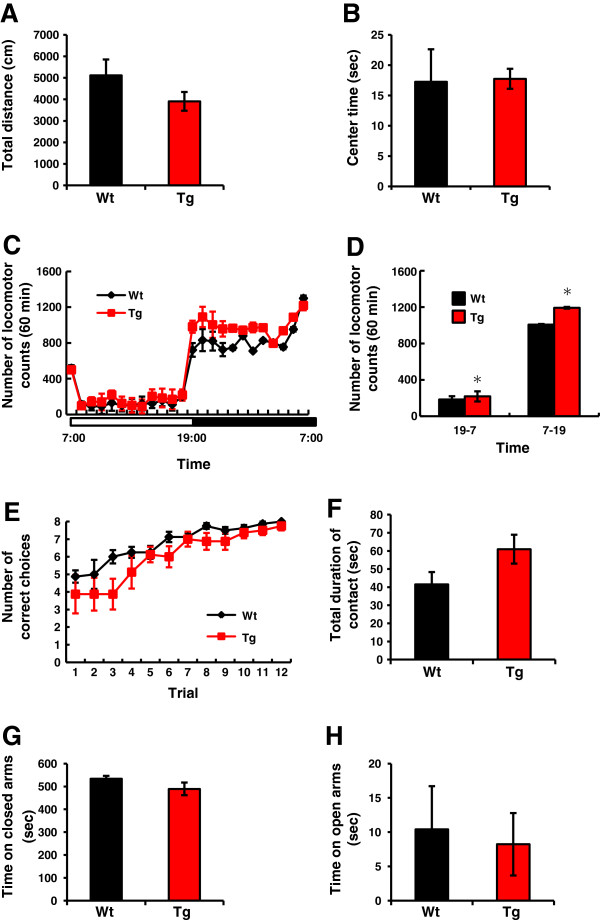
**Behavioral effects of Vldlr overexpression.** Adult (2 to 3 months) rats were used for all behavioral tests. (**A**,**B**) Open-field test. There was no significant difference between transgenic (Tg) and wild-type (Wt) rats in total distance moved (**A**) or time spent in the central area (**B**), *n* = 10 per genotype. (**C**,**D**) Locomotor activity in the home cage. Dark- (19:00 to 07:00) and light- (07:00 to 19:00) phase locomotor activity (**C**). Transgenic rats were significantly more active than Wt rats (*P* < 0.05, repeated measures ANOVA, *n* = 10 or 11 per genotype). Mean dark- and light-phase locomotor activity (**D**). Transgenic rats were significantly more active than Wt rats in both phases (* *P* < 0.05, unpaired Student’s *t*-test, *n* = 8 to 10 per genotype). (**E**) Radial maze test. Number of correct choices, among the first eight choices, during learning. Transgenic rats made fewer correct choices than Wt rats did (*P* = 0.091, repeated measures ANOVA, *n* = 8 per genotype). (**F**) Social interaction test. There was no significant difference between Tg and Wt rats for the total duration of contact. *n* = 10 per genotype. (**G**,**H**) Elevated plus maze test. There was no significant difference between Tg and Wt rats for the time spent in the closed arms (**G**) or the open arms (**H**). *n* = 10 per genotype. Error bars represent mean ± SEM.

We evaluated the effects of increased Vldlr on rat behavior. Adult (2 to 3 months) rats were used for all behavioral tests. First, we used the open-field test to investigate basic behaviors and exploratory locomotor activity in a novel environment. The total distance moved was evaluated as motor activity, and the time spent in the central area was evaluated as exploratory behavior. We did not find any significant difference in total distance moved (Figure 4A) or time spent in the center (Figure 4B), between Tg and Wt rats. Secondly, we measured spontaneous locomotor activity in a familiar home cage. Activity was significantly increased in Tg rats compared with Wt rats (*P* < 0.05, repeated measures ANOVA; Figure 4C). Increased activity was seen in both the dark and light phases (*P* < 0.05; Figure 4D), and Tg rats were hyperactive all the time. Thirdly, we assessed spatial working memory using an eight-arm radial maze. The time course of the number of correct choices among the first eight choices is shown (Figure 3E). Transgenic rats showed a trend toward fewer correct choices than Wt rats (*P* = 0.091, repeated measures ANOVA; Figure 4E). Finally, we conducted the social interaction test and elevated plus maze test to evaluate sociality and anxiety, respectively. We found no significant difference between Wt and Tg rats (Figure 4F–H).

## Discussion

We have developed a rat model in which the *Vldlr* transgene is driven by a strong ubiquitous promoter, to test the *in vivo* neurological effects of Vldlr overexpression. Vldlr overexpression changed downstream reelin signaling in Tg rats, as determined by reduced *Dab1* expression. Moreover, Tg rats showed abnormalities in *Vldlr* and *Dab1* mRNA expression, similar to those detected in post-mortem brain from patients with autism [30]. We found that spontaneous locomotor activity was significantly increased, and a tendency toward impaired memory function in Tg rats, without changes in histology.

In Vldlr knockout mice, the most remarkable histological phenotype is a smaller and less foliated cerebellum, with slight abnormalities found in the hippocampus and neuronal layers of the cerebral cortex [44]. In contrast, in Vldlr-Tg rats, there were no histological abnormalities, even in the cerebellum. This suggests that a 1.5-fold increase in Vldlr may not be sufficient to affect brain morphology.

The mechanism for the hyperactivity in the Tg rats is unclear. It has been reported that the SH3-domain kinase binding protein 1 (Sh3kbp1), which is involved in receptor endocytosis, binds Dab1, a downstream molecule in reelin signaling. The resulting interaction may play a role in the endocytosis of reelin receptors including Vldlr, and cause downregulation of reelin signaling [45]. Interestingly, Sh3kbp1 knockout mice display hyperactivity [46]. Additionally, they display abnormally high levels of dopamine and D2 dopamine receptors in the striatum [46]. Similarly, several studies have reported that reelin signaling is involved in dopamine receptor expression in the striatum [10,11]. Although there has been no report of Vldlr expression in Sh3kbp1 knockout mice, it is feasible that membrane expression of Vldlr is upregulated, resulting in facilitation of the dopaminergic system and hyperactivity in the mice. This suggests that Vldlr regulates the dopaminergic system, and may cause the hyperactivity observed in the Tg rats.

The spontaneous social interaction test that we used in this study is a standard test used to measure social interaction between animals. Using this test enabled us to examine other social behaviors, such as aggression, without performing additional behavioral tests. In the course of our testing, we ascertained aggressive behavior, and found Vldlr-Tg rats not to be aggressive (data not shown). We did not apply other tests to measure social behavior, such as a social novelty test, in depth, so there remains the possibility that Vldlr-Tg rats may show differences in some domains of social behavior.

## Conclusions

Several reports have implicated reelin signaling in the etiology of neurodevelopmental and psychiatric disorders [27-35]. However, patients with these disorders do not show striking neuroanatomical aberrations. For example, although post-mortem and structural magnetic resonance imaging have highlighted the frontal lobes, amygdala, and cerebellum as pathologically affected in autism, there is no clear and consistent pathology for this disorder [47]. Notably, hyperactivity and working memory impairment are symptoms observed in neurodevelopmental and psychiatric disorders, including autism [48]. Therefore, our findings of hyperactivity and memory impairment in Vldlr-Tg rats are significantly relevant, from a clinical point of view.

## Abbreviations

Dab1: Disabled homolog 1; EGFP: Enhanced green fluorescent protein; IRES: Internal ribosome entry site; Lrp8: Low-density lipoprotein receptor-related protein 8; NeuN: Neuronal nuclei; PBS(−): Phosphate-buffered saline without Mg^2+^ or Ca^2+^; qRT-PCR: Quantitative reverse transcription-polymerase chain reaction; SDS: Sodium dodecyl sulfate; Sh3kbp1: SH3-domain kinase binding protein 1; Tg: Transgenic; Vldlr: Very-low-density lipoprotein receptor; Wt: Wild-type.

## Competing interests

The authors declare that they have no competing interests.

## Authors’ contributions

HM, KI, and NM designed the study. KI, HM, IT, AA, MMV, CS, and KN generated the Vldlr-Tg rats. KI, TT, KS, Y Iwata, TW, and YK performed the histology. KI, NI, TM, Y Ishibashi, Y Ichitani, and KY performed the behavioral tests. KI, HM, NI, and NM prepared the manuscript. All authors read and approved the final manuscript.

## Supplementary Material

Additional file 1 Figure S1 Neuron number and thickness of cortical layers. (A) Quantification of NeuN-positive neurons in cortical layers (I, II/III, IV, V, and VI). (B) Quantification of cortical layer thickness (I, II/III, IV, V, and VI). In both A & B, results are presented as a percentage of controls (wild-type rats). Errors bars represent mean ± SEM (*n* = 4 per genotype). Click here for file

Additional file 2 Figure S2 Neuroanatomical characterization of VLDLR transgenic (Tg) rats at P0. Histology of the cortex, hippocampus, and cerebellum in wild-type (Wt) and transgenic (Tg) rats at P0. Panels show Nissl-stained sagittal sections. Results are representative of four independent experiments. Scale bar, 500 μm. Click here for file

Additional file 3 Supplementary methods Quantitative analysis. For the cell count in cortical layers, and measurement of cortical layer thickness, corresponding areas were sampled randomly, according to the optical fractionator method. NeuN-positive neurons were counted in three-dimensional counting frames. The number of cells was counted in the right hemisphere of every 16th section. Thickness of the individual cortical layers (I, II/III, IV, V, VI; Figure 3B) were measured in the right hemisphere of every 16th section. Sectioning, cell counting, and measurement of cortical layer thickness were performed by separate investigators who were blinded with respect to the animals’ genotype. Data were analyzed by unpaired Student’s t tests. Click here for file
