# Supplementary figures and images for: Vldlr overexpression causes hyperactivity in rats
Source: Mol Autism. 2012 Oct 30;3:11. doi: 10.1186/2040-2392-3-11 (PMC3533969; doi:10.1186/2040-2392-3-11)

# Supplementary Fig. 1

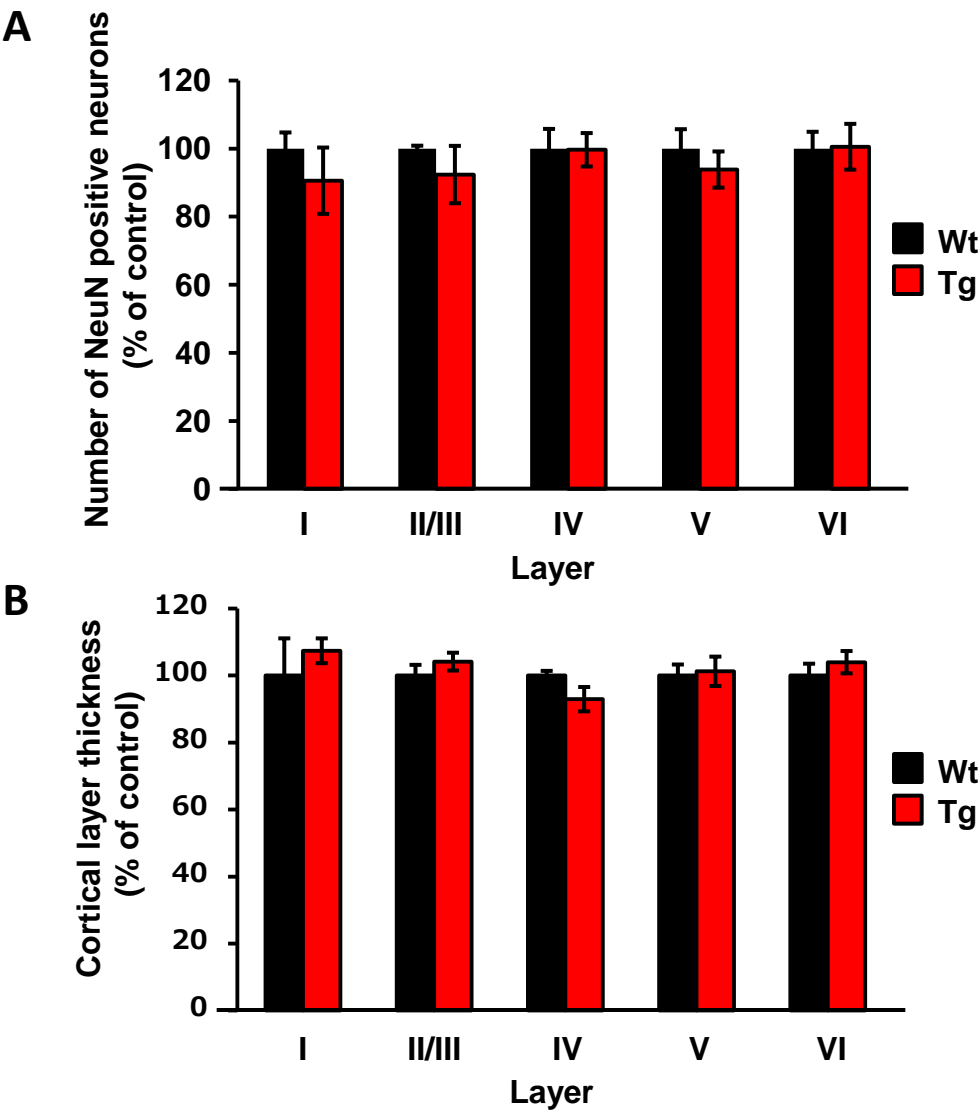

Supplement: Additional file 1 Figure S1 — Neuron number and thickness of cortical layers. (A) Quantification of NeuN-positive neurons in cortical layers (I, II/III, IV, V, and VI). (B) Quantification of cortical layer thickness (I, II/III, IV, V, and VI). In both A & B, results are presented as a percentage of controls (wild-type rats). Errors bars represent mean ± SEM (n = 4 per genotype). [file 2040-2392-3-11-S1.pdf]

Supplementary Fig. 2

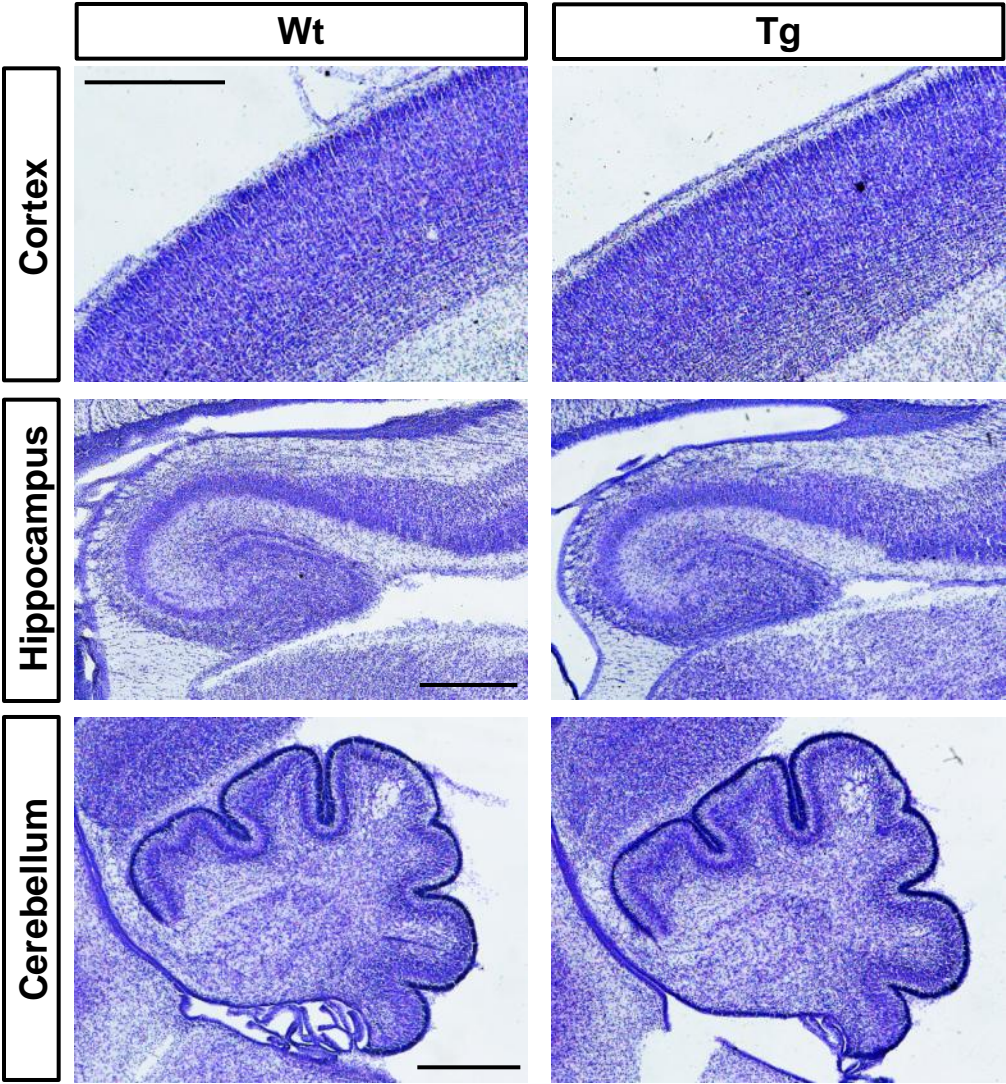

Supplement: Additional file 2 Figure S2 — Neuroanatomical characterization of VLDLR transgenic (Tg) rats at P0. Histology of the cortex, hippocampus, and cerebellum in wild-type (Wt) and transgenic (Tg) rats at P0. Panels show Nissl-stained sagittal sections. Results are representative of four independent experiments. Scale bar, 500 μm. [file 2040-2392-3-11-S2.pdf]
