# Supplementary material for: Vldlr overexpression causes hyperactivity in rats
Source: Mol Autism. 2012 Oct 30;3:11. doi: 10.1186/2040-2392-3-11 (PMC3533969; doi:10.1186/2040-2392-3-11)
Supplement: Additional file 3 Supplementary methods — Quantitative analysis. For the cell count in cortical layers, and measurement of cortical layer thickness, corresponding areas were sampled randomly, according to the optical fractionator method. NeuN-positive neurons were counted in three-dimensional counting frames. The number of cells was counted in the right hemisphere of every 16th section. Thickness of the individual cortical layers (I, II/III, IV, V, VI; Figure 3B) were measured in the right hemisphere of every 16th section. Sectioning, cell counting, and measurement of cortical layer thickness were performed by separate investigators who were blinded with respect to the animals’ genotype. Data were analyzed by unpaired Student’s t tests. [file 2040-2392-3-11-S3.doc]

Supplementary methods

Quantitative analysis

For the cell count in cortical layers, and measurement of cortical layer thickness, corresponding areas were sampled randomly, according to the optical fractionator method. NeuN positive neurons were counted in 3-dimensional counting frames. The number of cells were counted in the right hemisphere of every 16th section. Thickness of the individual cortical layers (I, II/III, IV, V, VI; Fig. 3B) were measured in the right hemisphere of every 16th section. Sectioning, cell counting and measurement of cortical layer thickness were performed by separate investigators who were blinded with respect to the animals’ genotype. Data were analysed by unpaired t-tests.
